# Myocarditis Post Moderna Vaccination: Review of Criteria for Diagnosis

**DOI:** 10.7759/cureus.19633

**Published:** 2021-11-16

**Authors:** Jocelyn McCullough, Joseph P McCullough, Giridhar Korlipara, Alan Kaell

**Affiliations:** 1 Medicine, Zucker School of Medicine at Hofstra/Northwell, Hempstead, USA; 2 Hospital Medicine, Zucker School of Medicine at Hofstra/Northwell, Hempstead, USA; 3 Interventional Cardiology, Zucker School of Medicine at Hofstra/Northwell, Hempstead, USA; 4 Internal Medicine, Zucker School of Medicine at Hofstra/Northwell, Hempstead, USA

**Keywords:** endomyocardial biopsy, incident, centers for disease control and prevention (cdc), post vaccination myocarditis, covid vaccine-induced myocarditis

## Abstract

Case reports of myocarditis post-coronavirus disease 2019 (COVID-19) mRNA vaccination have not uniformly reported long-term follow-up beyond 90 days. We present a 23-year-old male who is typical of a patient presenting with myocarditis post-COVID-19 mRNA-1273 Moderna vaccination (young males, onset several days after second dose of the mRNA vaccine, and excellent short term complete recovery). Follow-up at 128 days revealed no residual sequelae in our patient. Although a definitive diagnosis of myocarditis requires an endomyocardial biopsy (EMB), diagnosis is usually made clinically and with imaging in most clinical settings unless part of an approved research protocol or if indicated clinically. We recommend active surveillance and reporting for myocarditis post mRNA vaccination and even consider reporting those with symptom onset beyond 90 days.

## Introduction

Myocarditis primarily involves the inflammation of the myocardium and can also affect the nearby structures such as the epicardium and pericardium [1]. The clinical presentation can vary from asymptomatic to severe chest pain with signs of heart failure and arrhythmia. The etiologies are various: infections (i.e., viruses, bacteria), autoimmune conditions, toxic substances, and some vaccines. Since June 2021, myocarditis has been reported in young patients who have received their second dose of coronavirus disease 2019 (COVID-19) mRNA vaccination [2-7]. As typical of other cases reported in this literature, our patient did not require endomyocardial biopsy (EMB) for diagnosis. The recent New England Journal of Medicine (NEJM) correspondence that reported one autopsy case and one EMB case is discussed [8]. We also review the three main resources for guidance and expert opinion on the matter of myocarditis diagnosis which include a 2007 scientific joint statement from the American Heart Association/American College of Cardiology (AHA/ACC) and European Society of Cardiology (ESC) [9], the 2013 ESC Working Group on Myocardial and Pericardial Diseases (ESC Working group) guidelines [1], and the more recent 2020 scientific statement from the AHA/ACC [10], focusing on the role of EMB. We compare the CDC case definition criteria to be used in the upcoming survey of patients reported through the Vaccine Adverse Event Reporting System (VAERS) and Vaccine Safety Datalink (VSD) programs to the Brighton Collaboration case definitions.

## Case presentation

A 23-year-old Caucasian male with a history of exercise-induced asthma presented to the emergency department complaining of left-sided chest pain which started two days after receiving the second dose of the mRNA-1273 Moderna vaccine. The patient described the pain as sharp, intermittent with radiation to the left upper back and left arm with 10/10 severity and worsening with deep inspiration. Fever and chills were also present. The patient did not report any recent history of tick bites, upper respiratory symptoms, paroxysmal nocturnal dyspnea (PND), orthopnea, arthralgias or rashes. 

On physical examination the patient was in no distress, with normal vital signs, normal S1/S2 heart sounds without any murmurs, rubs, or gallops and no jugular vein distention (JVD). There was no palpable tenderness of the chest wall. The lungs were clear to auscultation. There was no pitting edema in the lower extremities. 

**Figure 1:**
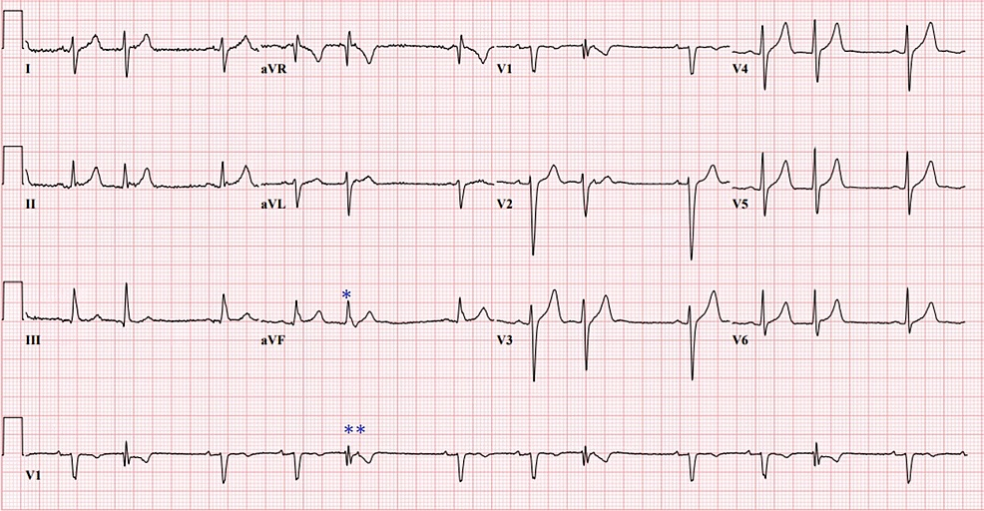
Patient's Electrocardiogram on Presentation *Upward facing QRS in I, AVF indicating rightward axis deviation with left posterior fascicular block **Premature atrial contraction (PAC) in trigeminy

Diagnostic testing revealed elevated troponin T of 475ng/L (<22ng/L) which trended upward reaching a peak of 910ng/L (<22ng/L). Initial electrocardiogram (ECG) showed right axis deviation with left posterior fascicular block without any ST elevations as well as premature atrial contractions (PACs) in trigeminy (Figure 1). A bedside ultrasound showed trace pericardial effusion. CT angiography (CTA) of the chest was negative for pulmonary embolism (PE). Lyme serology, antinuclear antibodies (ANA) and respiratory viral panel were negative and thyroid stimulating hormone (TSH) was normal. Pertinent leukocytosis of 11.09 K/ul (3.8-10.5 K/Ul) with absolute neutrophil count of 8.09 K/uL, elevated erythrocyte sedimentation rate (ESR) of 37mm/hr (0-15mm/hr), c-reactive protein (CRP) of 11.6mg/L (<4.0mg/L). Urine toxicology was positive for recreational marijuana drug use but negative for cocaine use.

Transthoracic echo (TTE) revealed abnormal motion and increased thickening of the septal wall with preserved ejection fraction (EF) of 65% and normal diastolic function (Video 1). Trace pericardial effusion was also noted. Based on the patient’s clinical presentation, ECG, cardiac markers and TTE findings a presumptive diagnosis of peri-myocarditis was made.

**Video 1:** Four chamber view of the patients initial echocardiography on presentation.

We did not pursue cardiac MRI since the patient had clinically improved within 48 hrs. He received aspirin 325 mg once followed by indomethacin 50mg twice a day and discharged on day three to complete a total of two weeks indomethacin and three months of colchicine 0.6mg daily. Complete resolution of his symptoms, normalization of troponins and ECG, was demonstrated within two weeks during follow up. At his 60-day follow-up visit, TTE confirmed resolution of the wall motion abnormality and pericardial effusion and he remains completely symptom free at 128 days.

## Discussion

Two recent independent retrospective studies attempt to ascertain the incidence of myocarditis following the BNT162b2 vaccine [11,12]. The reports differ in their case definitions. One utilizes the the Brighton Collaboration definition (Table 1) and the other uses CDC case definition of myocarditis following vaccination, originally developed for smallpox and influenza cases selected (Table 2). The two studies also differ as to when the onset of myocarditis was captured after the vaccination, as well as varying methodologies of chart identification and data extraction. Importantly, the two studies combined reported only three EMB were performed in 200 cases of myocarditis post-vaccination (1/54 cases using CDC criteria plus 2/136 cases using Brighton). 

**Table 1:** Brighton Collaboration Criteria ECG: Electrocardiogram; CK-MB: Creatine kinase - MB; CRP: C-reactive protein; ESR: Erythrocyte sedimentation rate; PAC: Premature atrial contraction; PVC: Premature ventricular contraction

Brighton Collaboration Criteria	
Definitive Diagnosis	Histopathological examination of myocardial tissue showing myocardial inflammation or elevated cardiac marker: Troponin T/I
	And
	Abnormal cardiac MRI with at least 1 of the following: Edema on T2 weighted study or late gadolinium enhancement on T1 with an increased ratio between myocardial and skeletal muscle (of non-ischemic origin) or Abnormal echo findings with at least 1 of the following: New focal or diffuse left/right ejection fraction, Wall motion abnormalities, Abnormality in global systolic/diastolic function, Ventricular dilation or change in wall thickness
Probable Case	Cardiac Symptoms at least 1 of the following: Acute chest pain/pressure, palpitations, dyspnea after exercise/rest, diaphoresis, sudden death or Nonspecific symptoms at least 2 of the following: Fatigue/dizziness/syncope, cough, edema, abdominal pain or infants and young kids at least 2 of the findings: Irritability, poor feeding, tachypnea
	And
	Testing supporting diagnosis (Biomarkers, Echocardiogram and ECG): Troponin I/T and CK-MB, Echo findings with at least 1 of the following: New focal or diffuse left/right ejection fraction, wall motion abnormalities, abnormality in global systolic/diastolic function, ventricular dilation, change in wall thickness, intracavitary thrombus or ECG abnormalities that are new/and or normalize on recovery (at least one of the following): paroxysmal/sustained atrial or ventricular arrhythmia, av nodal conduction delays or intraventricular conduction delays, continuous ambulatory ECG which show's atrial/ventricular ectopy
	And
	No alternative diagnosis
Possible Case	Cardiac Symptoms at least 1 of the following: Acute chest pain/pressure, palpitations, dyspnea after exercise/rest, diaphoresis, sudden death or Nonspecific symptoms at least 2 of the following: Fatigue/dizziness/syncope, cough, edema, abdominal pain or Infants and young kids at least 2 of the findings: Irritability, poor feeding, tachypnea
	And
	Biomarkers showing inflammation (at least 1 of the following): Elevated CRP, ESR, D-dimer
	And
	Non-specific ECG: ECG abnormalities that are new/and or normalize on recovery (at least one of the following): ST segment or T wave abnormality (elevation and inversion), PAC’s/PVC’s
	And
	No alternative diagnosis

**Table 2:** CDC Case Definition of Myocarditis ECG: Electrocardiogram; LV: Left ventricular; CMR: Cardiac magnetic resonance imaging

CDC Case Definition of Myocarditis	
Suspected Case	Dyspnea, palpitations, or chest pain of probable cardiac origin with either one of the following: A. ECG abnormalities beyond normal variants, not documented previously including: ST segment /T wave abnormalities, Paroxysmal or sustained atrial/ventricular arrhythmia, Atrioventricular nodal dysfunction delay's/ intraventricular conduction defects, Continuous ambulatory ECG monitoring that detects frequent atrial or ventricular ectopy B. Focal or diffuse depressed LV function of indeterminate age identified by an imaging study
Probable Case	Meets criteria for suspected myocarditis, in the absence of other likely cause of symptoms, in addition to one of the following: Elevated cardiac enzymes (troponin I, troponin T or creatine kinase-MB) , New onset or increased degree of severity of focal or diffuse depressed LV function by imaging, Abnormal imaging findings indicating myocardial inflammation (CMR with gadolinium, gallium 67 scanning, antimyosin antibody scanning)
Confirmed Case	Elevated cardiac enzymes (troponin I, troponin T or creatine kinase-MB), New onset or increased degree of severity of focal or diffuse depressed LV function by imaging, Abnormal imaging findings indicating myocardial inflammation (CMR with gadolinium, gallium 67 scanning, antimyosin antibody scanning)

Previously, two case reports included histopathological confirmed myocarditis (patient 1, a 45-year-old woman who received endomyocardial biopsy and patient 2, a 42-year-old man who had autopsy revealing myocarditis) [8]. Both patients met the ESC criteria for myocarditis, however, only patient 2 met the AHA/ACC criteria for pursuing EMB. In patient 1, biopsy is not likely to have played a role in her outcome given that she was discharged from hospital in seven days with a recovered ejection fraction to 60% and had clinical resolution of symptoms after 17 days [9]. This letter to the editor does not otherwise specify why EMB was performed on patient 1, likely due to word count limitations. A reply, however, from the author (personal communication) stated that the biopsy was performed due to acute heart failure with hemodynamic compromise. 

So, when should clinicians consider EMB in patients presenting with myocarditis post-mRNA vaccination? In 2007 the AHA/ACC and European Society of Cardiology created a scientific statement for practitioners to use on guidance for the use of EMB in newly diagnosed/suspected myocarditis. Their statement included 14 clinical scenarios in which the diagnostic, prognostic, and therapeutic value of EMB could be estimated compared with procedure-related risks (not including the role of EMB in the post-cardiac transplantation setting). These scenarios were grouped according to level of evidence, Class I being the most evidence, Class IIa being where there is some diverging opinion and limited evidence, Class IIb being scenarios where generally there is consensus not to perform EMB [9]. The clinical scenarios are summarized in Table 3. 

**Table 3:** Clinical Scenarios where EMB is Recommended Based on Level of Evidence LV: Left ventricle; AV: atrioventricular; HCM: Hypertrophic cardiomyopathy; ARVC: arrhythmogenic right ventricular cardiomyopathy; EMB: endomyocardial biopsy

Clinical Scenarios where EMB is recommended based on level of evidence	
Class I	Fulminant heart failure <2 weeks: Dilated LV with ventricular arrhythmia or AV block or refractory to conventional therapy 2 weeks - 3 months duration
Class IIa	Heart failure>3 months and dilated LV with ventricular arrhythmia or AV block or refractory to conventional therapy within 1-2 weeks, selected cardiac masses not myxomas, dilated LV with eosinophilia, restrictive cardiomyopathy or suspected anthracycline toxicity not diagnosed by imaging.
Class IIb	Heart failure>3 months and dilated LV without ventricular arrhythmia or AV block responds to usual therapy in 1-2 weeks duration, unexplained HCM, unexplained ARVC, unexplained Ventricular arrhythmia

In 2013 the European Society of Cardiology (ESC) Working Group on Myocardial and Pericardial diseases proposed guidelines for new diagnostic criteria to reinforce the diagnosis of myocarditis in suspected patients [1]. They go on to additionally define the criteria for classifying patients as having a ‘high clinical suspicion’ for myocarditis as outlined in Table 4. 

**Table 4:** ESC Working Groups Diagnostic Criteria for Clinically Suspected Myocarditis ESC: European Society of Cardiology; ECG: Electrocardiogram; AV: Atrioventricular; CMR: Cardiac magnetic resonance imaging; LV: Left ventricular; RV: Right ventricular; CAD: Coronary artery disease

ESC Working Groups Diagnostic Criteria for Clinically Suspected Myocarditis	
Clinical Presentation	Acute chest pain, New onset or worsening of dyspnea at rest/with exertion (up to 3 months), Subacute/chronic worsening of dyspnea at rest/with exertion (> 3 months), Palpitations, unexplained arrhythmia/syncope, sudden death, Unexplained cardiogenic shock
Diagnostic Criteria	ECG features: AV blocks, ST/T wave changes, sinus arrest, ventricular tachycardia, atrial fibrillation, reduced R wave height, intraventricular conduction delay (widened QRS complex), abnormal Q waves, low voltage, frequent premature beats, supraventricular tachycardias. Myocardial markers: elevated troponins. Functional/structural abnormalities on cardiac imaging (CMR/echo/angiography): New or unexplained LV/RV structure or function abnormalities such as regional wall motion or global systolic or diastolic dysfunction (with or without ventricular dilatation, increased wall thickness, pericardial effusion, or endocavitary thrombi). Tissue characterization on Cardiac Magnetic Resonance (CMR) imaging: Edema or Late gadolinium enhancement (LGE) as seen in classic myocarditis
	Clinical suspicion is high if ≥1 clinical presentation and ≥1 diagnostic criteria. Additionally, it must be in the absences of angiographically detectable CAD, pre-existing CAD or extra cardiac causes that could otherwise explain the clinical picture

These ESC guidelines strongly recommend that EMB be performed on all patients with suspected myocarditis so that proper therapy can be directed, particularly if considering antiviral vs immunosuppressive treatments [1]. The ESC working group position is that increasing the rate of EMB in patients presenting with myocarditis will target the subset of patients that progress to dilated cardiomyopathy and have an associated worse prognosis. EMB in these patients would help to direct diagnosis, prognosis and therapeutic options for these patients potentially preventing progression of their disease state. If EMB is performed they recommend performing histology, immunochemistry, viral polymerase chain reaction (PCR). Additionally, three samples 1-2 mm in size fixed in 10% formalin at room temp for light microscope and additional samples taken and frozen for viral PCR. While complications exist (the most worrisome cardiac tamponade) they propose that the rates are low if performed by experienced experts [13]. Yet, medical centers may have limited resources to perform these tests safely, precisely, and reliably. 

In stark contrast to the 2013 ESC guidelines, the AHA released a scientific statement in 2020 that narrows the scope of patients to a select group of patients that EMB would offer the greatest potential change in prognosis and management [10]. This statement addresses the role of EMB as a diagnostic tool in patients with ‘fulminant myocarditis’. Even in these patients with diffuse inflammation of the myocardium causing hemodynamic instability,arrhythmia and even death the patchy pattern of inflammation increases the sampling error associated with EMB [10]. This 2020 statement reaffirms the position of prior scientific statements from the AHA/ACC regarding the indications for EMB in addition to those with rapidly progressing heart failure as seen in Table 5. 

**Table 5:** AHA/ACC 2020 Recommendation for Use of EMB EMB: endomyocardial biopsy; AHA/ACC: American Heart Association/American College of Cardiology

AHA/ACC 2020 recommendation for use of EMB
EMB is recommended for the following patients with an unexplained acute cardiomyopathy:
Requiring inotropic or mechanical circulatory support, Mobitz type II or higher heart block, Sustained or symptomatic ventricular tachycardia, Failure to respond to guideline based medical management within 2 weeks

Currently, the CDC does not provide guidance for confirming a diagnosis of patients with high clinical suspicion for myocarditis. They do refer to a 2021 scientific statement from AHA/ACC for the follow-up care and monitoring of these patients in the outpatient setting. In that statement the AHA/ACC favors the use of cardiac MRI (CMRI) over EMB in confirming the diagnosis of myocarditis. They explain that increased use of CMRI has revealed that myocarditis occurs most often in the left ventricular free wall (which is often not reached with standard EMB technique) [14]. 

As it currently stands the necessity for EMB in patients with whom we suspect myocarditis secondary to the COVID-19 mRNA vaccines is in question since the expectation for patients is complete recovery with excellent short-term prognosis [2-7]. 

Although cases of post-vaccine myocarditis following mRNA 1273 Moderna are described, a similar analysis of incidence, clinical experience, and short-term prognosis could not be found. Other than the storage requirements and the 28 days between the two doses, we anticipate that there may be similar studies and results forthcoming. 

On reviewing other case studies published in Cureus since May of 2021 we do not find that EMB has been performed for confirmation of diagnosis or for prognostic purposes. This includes two such case reports of five patients and another with eight patients presenting that all had varying clinical symptoms all of whom were treated and discharged from an inpatient setting between two to seven days with excellent short-term recovery [2,4]. All these patients met the diagnostic criteria for ‘high clinical suspicion’ of myocarditis as put forth by the ESC working group (Table 4).

Thus far, we have yet to see the long-term (>90 day) survey follow-up of post-COVID-19 mRNA vaccination myocarditis cases. The CDC is planning to roll out a survey to those meeting defined CDC myocarditis post-mRNA vaccination reported via the VAERS program [15-17]. The U.S. Food and Drug Administration (FDA) requires healthcare professionals to report to the Vaccine Adverse Event Reporting System (VAERS) certain serious adverse events and hospitalizations that occur after COVID-19 vaccination [16,17]. Those patients who meet the CDC’s case definition for myocarditis following mRNA COVID19 vaccination and who have been reported to the VAERS by their HCP are being contacted by the CDC to participate in voluntary surveillance so that outcomes > 90 days from this side effect can be recognized. As of October 6th, 2021, the CDC has confirmed 1640 cases of myocarditis/pericarditis through VAERS and through case review confirmed 926 cases [18]. According to the CDC the typical patients are young males who have received their second dose of the mRNA vaccine with symptoms generally starting several days after receiving the vaccine. This is similar to the incidence data from Israel. Each study varies in methodology. 

In the USA, CDC will contact people with myocarditis reported to VAERS after at least 90 days have passed since myocarditis symptoms began. This outreach is anticipated to begin in fall of 2021. The CDC case definition for suspected, probable, and definitive myocarditis, established from the 2003 criteria used for smallpox and influenza vaccine reports of myocarditis is seen in Table 2.

Healthcare providers are strongly encouraged to report to VAERS if A) Any adverse event that occurs after the administration of a vaccine licensed in the United States, whether it is or is not clear that a vaccine caused the adverse event or B) Vaccine administration errors. We encourage all suspected USA cases of mRNA vaccine related myocarditis be reported through the VAERS program via the following link: https://vaers.hhs.gov/reportevent.html as well as those mandated by law. 

## Conclusions

Providers evaluating patients (particularly male patients less than 30 years of age) presenting with chest pain after recent immunization with COVID mRNA vaccine should have a high suspicion for myocarditis/pericarditis. As most cases are self-limited, EMB does not appear to be currently necessary for clinical diagnosis, therapeutic choices, or prognosis. We propose to continue to follow the 2020 ACC/AHA guidelines on when EMB should be performed. Patients with myocarditis and their providers should be reporting these to VAERS. To monitor for any potential long-term side effects of myocarditis induced by the COVID-19 mRNA vaccines we encourage participation in the upcoming CDC survey requests for follow-up information after greater than 90 days.
